# Inference on spatial heterogeneity in tumor microenvironment using spatial transcriptomics data

**DOI:** 10.1002/cso2.1043

**Published:** 2022-08-11

**Authors:** Antara Biswas, Bassel Ghaddar, Gregory Riedlinger, Subhajyoti De

**Affiliations:** Rutgers Cancer Institute, Rutgers the State University of New Jersey, New Brunswick, New Jersey, USA

**Keywords:** cancer, heterogeneity, multivariate analysis, spatial transcriptomics, tissue microenvironment

## Abstract

In the tumor microenvironment (TME), functional interactions among tumor, immune, and stromal cells and the extracellular matrix play key roles in tumor progression, invasion, immune modulation, and response to treatment. Intratumor heterogeneity is ubiquitous not only at the genetic and transcriptomic levels but also in the composition and characteristics of TME. However, quantitative inference on spatial heterogeneity in the TME is still limited. Here, we propose a framework to use network graph-based spatial statistical models on spatially annotated molecular data to gain insights into modularity and spatial heterogeneity in the TME. Applying the framework to spatial transcriptomics data from pancreatic ductal adenocarcinoma samples, we observed significant global and local spatially correlated patterns in the abundance score of tumor cells; in contrast, immune cell types showed dispersed patterns in the TME. Hypoxia, EMT, and inflammation signatures contributed to intra-tumor spatial variations. Spatial patterns in cell type abundance and pathway signatures in the TME potentially impact tumor growth dynamics and cancer hallmarks. Tumor biopsies are integral to the diagnosis and clinical management of cancer patients; our data suggest that owing to intra-tumor non-genetic spatial heterogeneity, individual biopsies may underappreciate the extent of clinically relevant, functional variations across geographic regions within tumors.

## 1 | INTRODUCTION

While individual cell types have specialized functions, the tissue microenvironment provides a framework for an organized cellular activity to produce coordinated organ-level functions [1] and forms the basis for multicellular life. Tissue microenvironments tend to be self-organizing units, as observed during development, wound healing, and organoids [2]. In cancer, the tumor microenvironment (TME), which is comprised of the tumor, immune, stromal and extracellular compartments, is remodeled during tumorigenesis, and the TME in turn plays a key role in cancer progression, tissue invasion, metastasis, and response to treatment [3].

Heterogeneity and evolvability are hallmarks of all cancers, and intra-tumor heterogeneity is widespread not only at the genetic and transcriptomic levels but also in the composition and characteristics of TME. Spatial transcriptomics has been used to study a number of cancer types [4–8], revealing regional niches and heterogeneity, identifying spatially regulated compartments and genes, and localizing tumor-immune interfaces or lack thereof [9, 10]. However, efforts thus far to define spatial compartments in the TME have largely been observational, and quantitative inference on spatial heterogeneity in the TME is limited due to a number of factors. High throughput technologies for spatially annotated molecular profiling are developed only recently. Adjacent tissue regions are not independent data points for statistical analyses. Due to organ architecture and potential tissue distortions during sample preparation the patterns of cell-to-cell contacts in tissue contexts may be more relevant than Euclidean distances. To address these challenges, we propose a framework to use network graph-based spatial statistical models on spatially annotated molecular data to quantitatively examine modularity and spatial organization in the TME. Such models have been adopted in ecological modeling and allow us to incorporate geometric and context-dependent constraints. We apply the framework to spatial transcriptomics data to gain insights into spatial patterns and heterogeneity at the level of cell type abundance and pathway-level signatures in pancreatic cancer.

## 2 | RESULTS

**FIGURE 1:**
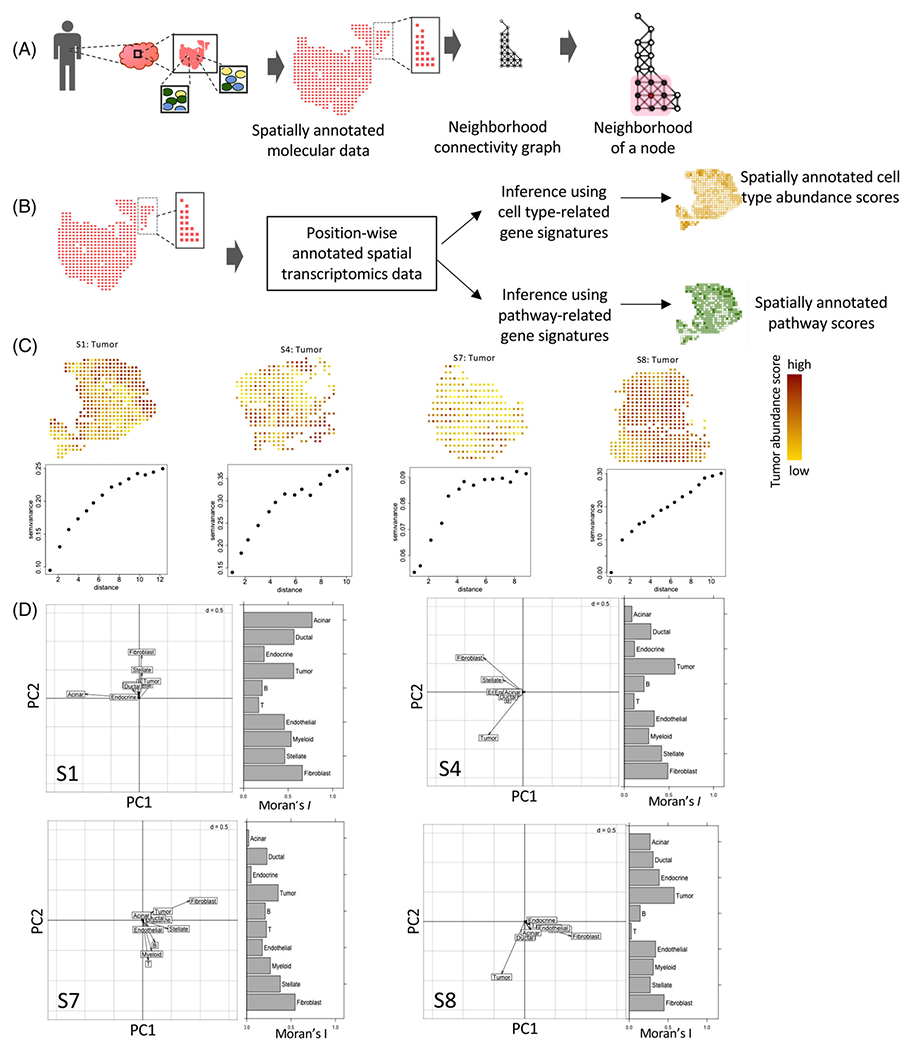
(A) A schematic representation showing a collection of tissue from a patient and spatial transcriptomics on a tissue section to profile the transcriptome of multiple spatially annotated units simultaneously. Based on the spatial annotation of the units, a neighborhood graph can be constructed. (B) A flowchart showing inference on cell type and pathway scores from spatial transcriptomic data using gene signatures. (C) Tumor cell abundance score from spatial transcriptomics data for the pancreatic ductal adenocarcinoma specimens. The variograms indicate the decay in correlation in tumor cell estimate score over distance in terms of the spatial units in the tumor microenvironment. (D) Multivariate spatial analysis showing joint variation in spatial localization of the cell types in the four samples. The spatial principal component analysis (sPCA) plot shows the loading of different cell types along the first two principal axes. Moran’s I indicate the extent of spatial autocorrelation coefficients of the cell types.

Emerging biomedical technologies allow the collection of high throughput molecular data from spatially annotated spots in tissues. We outline a framework to project spatially annotated molecular data onto a neighborhood connectivity graph, where each spatially annotated data point from a tissue sample is a node, and two adjacent nodes are connected by an edge (Figure 1A). The edges in the graph could be constructed based on any reasonable proximity criteria such as physical adjacency, Euclidean distance, Delaunay triangulation, and so forth, and/or based on additional constraints. Given tissue architectures and contours, and sample preparation techniques, some distance measures may be more relevant than the Euclidean distance in specific contexts. The network graph transformation retains spatial relationships among the spatially barcoded regions and allows the application of graph-based ecological statistics to examine aspects of tissue rheology in the TME while incorporating context-dependent constraints imposed by tissue architecture, biophysical characteristics, or experimental conditions.

We showcase the framework utilizing data obtained from spatial transcriptomics technologies which allow gene expression profiling of 10^2^–10^3^ annotated units from histological slides, each having a distinct X-Y coordinate-based barcode (Figure 1B); individual barcodes may have aggregated transcriptomic data from 10–100 cells - typically representing multiple cell types depending on the tissue context. As a case study, we analyzed spatial transcriptomics data for pancreatic ductal adenocarcinoma samples from Moncada et al. [5] and used marker gene expression signatures to estimate cell-type abundance scores of pancreatic tumor cells, as well as immune (myeloid, T cell, and B cell) and stromal cell types (fibroblasts, acinar, stellate and normal ductal cells) for each spatial barcode (see Methods; Figure 1B and S1).

First, we used Moran’s I, a measure of spatial autocorrelation, to assess whether cellular abundance correlates among spatially adjacent barcodes in the neighborhood graph. Results for four ‘representative’ samples are shown, and Supporting Information Figures present equivalent results for other samples. *I* values were positive (0.35–0.57) and statistically significant for tumor cell abundance scores in all the samples (Figure 1C and Figure S2). Using variogram analysis, we observed that semi-variance, a measure of distance-dependent decay in association, increased by 40–80% over a distance equivalent of 4–6 units (600-1000*μm*) - which is comparable to the typical size of pancreatic organoids observed in laboratory conditions (StemCell Technologies Inc., Doc #27088). We then jointly analyzed spatial abundance patterns of all cell types (Figure S1); apart from tumor cells, fibroblasts showed significantly high Moran’s I indicating spatial autocorrelation in all samples. In contrast, immune cell types were more dispersed and showed negligible spatial autocorrelation.

Next, we used spatial principal component analysis (sPCA) to examine regional variation in cell type compositions in each tumor histological slide (Figure 1D and Figures S1 and S2), while taking into consideration any potential spatial correlation in the neighborhood connectivity graph. Abundance scores of tumor cells and fibroblasts contributed to considerable loadings of the first two PCs of the sPCA – indicating that tumor cells and fibroblasts primarily dominate the patterns of spatial cell type heterogeneity in pancreatic tumors. Immune cell types had a minor contribution to the principal components (PCs), and the association between patterns of spatial abundance of tumor and T cells was consistently weak, suggesting a lack of concordance in spatial patterns of tumor and immune heterogeneity. These observations are consistent with reports that pancreatic tumors are typically immune cold [4, 5].

**FIGURE 2:**
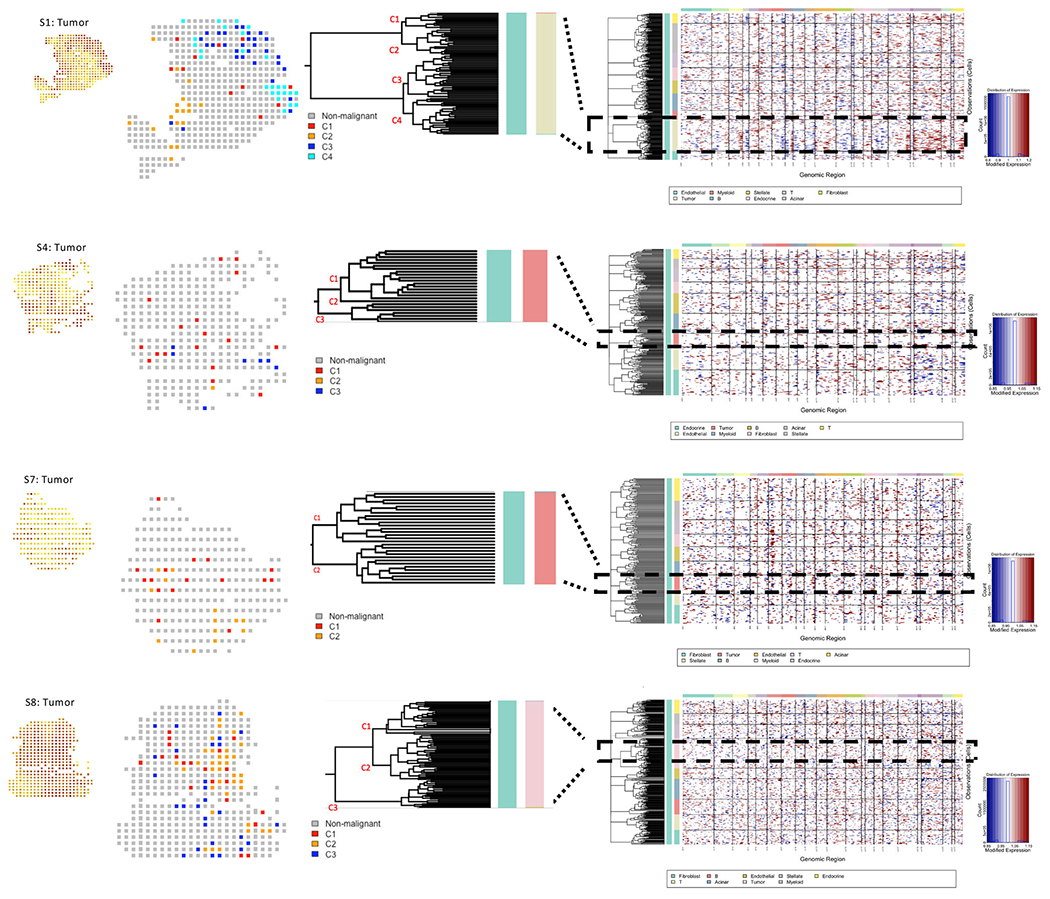
Copy number profiles estimated from spatial gene expression data for four pancreatic tumor samples. Hierarchical clustering of cells in each of the pancreatic tumor samples based on copy number profiles estimated using InferCNV, with each row corresponding to a cell, ordered by cell types, and clustered within each cell type by copy number patterns. Dashed rectangle reflects tumor-specific patterns and the zoomed-in dendrogram shows main tumor subclones, with visualization of spatial localization of the subclones and corresponding tumor abundance areas

To investigate spatial heterogeneity in terms of the clonal composition of tumor cells, we identified large-scale copy number variations (CNVs) in the tumor cells, and accordingly, clustered the spatially annotated regions into 2–4 major subclones (Figure 2 and Figure S3). Although we did not have access to actual samples for validation, the copy number and clonal architecture inferences appeared reasonable. For example, S1 and S4, which were obtained from different regions from the same tumor specimen, had the same CNV pattern, verifying that the same clusters across different sections had identical CNV characteristics. We found heterogeneity in terms of CNV-based clonal architectures, and spatial distributions of major subclones. For instance, C4, a minor subclone had a localized presence in S1, but not in S4. Likewise, C2, another subclone was common in S1 but rare in S4. Overall, spatial distributions of the subclones varied within and across tumors, but in most tumors, the major subclones overlapped, at least partly, in their spatial localization.

Next, we examined spatial patterns of cellular activities in the same samples. We computed enrichment scores for several pathways [11, 12] related to cancer hallmarks and tumor-immune interactions [13] for each spatial unit using a published approach [12] (Figure S4). Hypoxia showed consistent and significant spatial autocorrelation in all samples, suggesting that hypoxia signatures may be broadly distributed in TMEs. Cell cycle and apoptosis were more heterogeneous, potentially suggesting stochastic cell growth and death, both in tumor and non-malignant cell populations. Cell cycle activity was closely associated with tumor cell-rich regions and did not overlap with hypoxia. Spatial autocorrelation in EMT was considerably common in the samples; this might be due to composite effects of tumor growth, migration and other factors.

**FIGURE 3:**
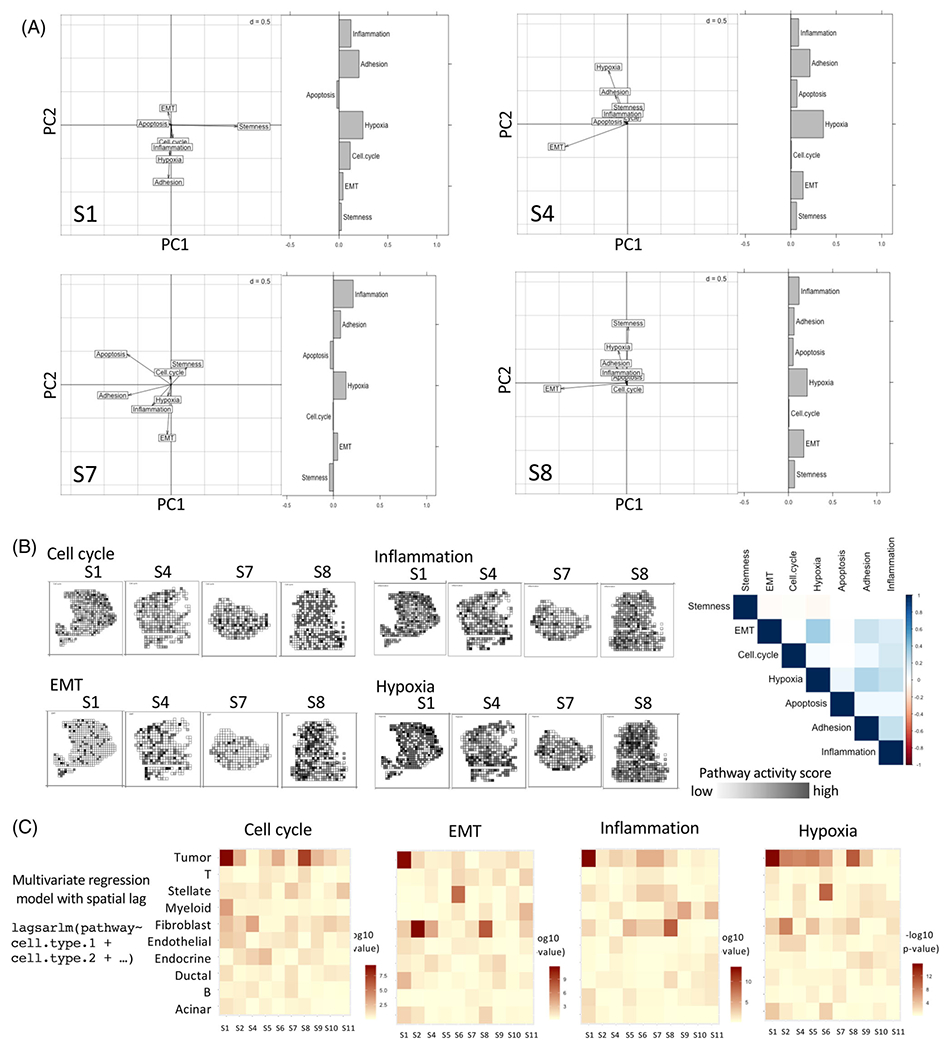
(A) Multivariate spatial analysis showing joint variation in spatial localization of the pathways associated with cancer hallmarks in the four samples. The spatial principal component analysis (sPCA) plot shows the loading of different pathway scores along the first two principal axes. Moran’s I indicate the extent of spatial autocorrelation coefficients of the pathways. (B) Representative pathways with high loadings are spatially presented for the tumor samples with a correlation plot for pathways associated with cancer hallmarks. The intensity of the black color indicates proportionally higher pathway-level activity (C) Pathway activity was modeled as a function of the estimated abundance of the cell types in the spatial transcriptomic data using multivariate regression with a spatial lag to account for spatial autocorrelation. Heatmap showing p-value associated with coefficients for the cell types in all pancreatic tumor samples. Rho and residual variance values are indicated in Table S4

Extending the sPCA approach to pathway-level signatures, we found that EMT and hypoxia were dominant features that consistently had the highest loading in the first two PCs – suggesting that EMT and hypoxia signatures contributed to most regional variations in pathway activities on the tissue slides (Figure 3A,B). Interestingly, although immune cells were heterogeneously distributed, the inflammation signature had substantial loading in multiple samples, typically along an axis orthogonal to EMT, indicating that inflammation signatures also contribute to regional variation in vivo.

Next, we modeled regional variation in the pathway activities based on regional abundances of the tumor, immune, and other cell types, using a multivariate regression model with a spatial lag to account for spatial autocorrelation along the neighborhood graph (Figure 3C and Figure S5). In tumors S1 and S4, EMT and cell cycle signatures were significantly associated with tumor cell abundance, while the effects were weaker in the other two samples. Inflammation was significantly associated with an abundance of immune (e.g., myeloid cells in S1) and tumor cells, but the effects varied between samples. Nonetheless, the proportion of variance in pathway-level scores estimated by spatial autoregressive parameters Rho and residual variance, explaining the abundance of different cell types was modest (Table S4). Therefore, there were substantial inter-tumor variations in the patterns of spatial heterogeneity in TME characteristics.

## 3 | DISCUSSION

Our analyses show that the transformation of spatially annotated omics data from tissue sections using neighborhood graphs and spatial multivariate modeling provides insights into the modularity and spatial heterogeneity of the TME. Spatially correlated patterns in tumor cell abundance might be due to clonal growth and moderate density-dependent migration of tumor cells [14]. In contrast, the immune landscape was more heterogeneous in these samples – which is in line with reports that immune microenvironments are more dynamic, pancreatic tumors are typically immunologically cold, and that immune evasion is common in pancreatic adenocarcinoma [15]. Pathway-level analysis indicated that hypoxia is widespread and spatially correlated in TME. In the sPCA analysis, EMT, hypoxia and inflammation explained regional variations, indicating that those might be among the key functional contexts shaping the pancreatic cancer microenvironment [3, 15, 16]. The lack of spatial autocorrelation observed for immune cells suggests that factors other than cell-type composition, EMT and hypoxia drive immune migration in tumors.

It is important to note the technical limitations for a balanced perspective. First, inference from spatial transcriptomics data can be biased by cell-type-specific biomarker selection, the effectiveness of deconvolution, regional variation in cell densities, and technical variations [10, 17]. Second, calling copy number alterations from spatial transcriptomic data is challenging. Third, without access to the original specimen, we were unable to estimate and validate relevant biophysical measurements from the spatial transcriptomics data. Tissue-specimens reflect a snapshot in time and do not directly track dynamic changes in tissue microenvironments. For the same reason, we did not extend the multivariate analysis to include tumor subclones. Fourth, our analysis was based on 2D spatial transcriptomic data, which lacked the 3D perspective, and our analyses involved relatively straightforward spatial autocorrelation and multivariate regression; future works will consider more advanced models that integrate multiple features to understand the organizing principles of the TME. Despite these limitations, spatial analysis of deconvoluted spatial transcriptomic data still provides the first quantitative metric by which to define a microenvironment and identify spatially dependent features.

Tumor biopsies are integral to the diagnosis and clinical management of cancer patients. Patterns of the tumor, immune and pathway-level spatial heterogeneity suggest that individual biopsies may underappreciate the extent of clinically relevant, functional variations at different levels, especially in immune phenotypes across the geographic regions within pancreatic tumors [18, 19]. However, liquid biopsies may address these limitations in the future [20, 21]. Lastly, our approach utilizing the neighborhood connectivity graph and corresponding spatial applications complements emerging resources to infer cellular connectomes [22–24] and the composition of tissue contexts [25–27] using single cell and spatial transcriptomics data at a different resolution to investigate spatiotemporal dynamics in healthy and diseased tissues.

## 4 | MATERIALS AND METHODS

### 4.1 | Spatial transcriptomics dataset

The spatial data for PDAC tumors were obtained from Moncada et al. [5]; each sample contained 243–996 spatially annotated barcodes, each capturing the aggregated transcriptomic makeup of potentially 20–70 cells in the tumor and adjacent normal tissue microenvironments. Each of the spatially barcoded spots on the array was 100 *μ*m in diameter and 200 *μ*m from center to center. These data were TP10K normalized, and cell-type abundance scores for each spatial barcode were calculated as the mean of expression of the cell-type specific marker genes on each respective barcode. The list of cell-type gene markers and cell types for each tumor sample is provided in Tables S1 and S2, respectively. The gene signature sets for the selected pathways were obtained from the MSigDB database [11]. For each spatial barcode, the pathway-level activity scores were determined based on the enrichment of the gene-set signatures within the expressed genes using AUCell [12] with default parameters (Table S3). Four representative samples- S1, S4, S7, and S8 are discussed in the main figures and the rest of the samples are in the Supporting Information Figures.

### 4.2 | Inference in clonal architecture

We used InferCNV [28] to estimate copy number status from spatial transcriptomic data in tumors and related other somatic cell types using ductal cells as reference cells, and use inferred copy number status to cluster the barcodes and annotate major subclones. We marked the major 2–4 dominant subclones, and annotated spatial barcodes with the dominant tumor subclones that occupied that position on the slide.

### 4.3 | Neighborhood connectivity graph

We define a neighborhood connectivity graph as a connected network, where each spatially annotated data point is a node, and two adjacent nodes are connected by an edge (Figure 1A). During analyzing spatial transcriptomics data, we connected adjacent spatially annotated spots with edges, after excluding those separated by empty spots. All edges in the neighborhood connectivity graph had equal weight.

### 4.4 | Variogram

The semivariogram *γ*(*d*) is half the mean squared difference between the values at points s1 and s2 separated by a distance *d*, which shows distance-dependent decay (or lack thereof) in a feature in the context of spatial interactions.

### 4.5 | Moran’s I

Moran’s spatial autocorrelation measure is defined as

$$I=\frac{N}{W}\frac{\sum_{i}\sum_{j}w_{ij}(x_{i}-\bar{x})(x_{j}-\bar{x})}{\sum_{i}{\left(x_{i}-\bar{x}\right)}^{2}}$$

where *N* is the total number of spatial units indexed with *i* and *j*; *x* is the random variable, in this case, a phenotype score for tissue microenvironment in the spatial units; $\bar{x}$ is the mean of *x*; *w_ij_* is a matrix of spatial weights and *w*_*ii*_ = 0; and *W* is the sum of all *w*_*ij*_.

### 4.6 | Spatial PCA and multivariate regression

Since spatially proximal entities are correlated, it may not be fair to consider individual observations (e.g., spatial units) as independent and use simple PCA and regression. Therefore, we used sPCA to assess phenotypic variation among the spatial barcodes using principal component analysis after taking into consideration the neighborhood graphs. Similarly, we used regression with a spatial lag model (lagsarlm), as implemented in the spatialreg R package, to perform multivariate regression.

## Supplementary Material

Supporting Materials

Supplementary Table 1

Supplementary Table 3

Supplementary Table 2

Supplementary Table 4

## Data Availability

The data that support the findings of this study are available from the corresponding author upon reasonable request.
