# Supplementary material for: Inference on spatial heterogeneity in tumor microenvironment using spatial transcriptomics data
Source: Comput Syst Oncol. Author manuscript; Available in PMC 2022 Sep 1. (PMC9410565; doi:10.1002/cso2.1043)
Supplement: Supporting Materials [file NIHMS1830146-supplement-Supporting_Materials.pdf]

S1

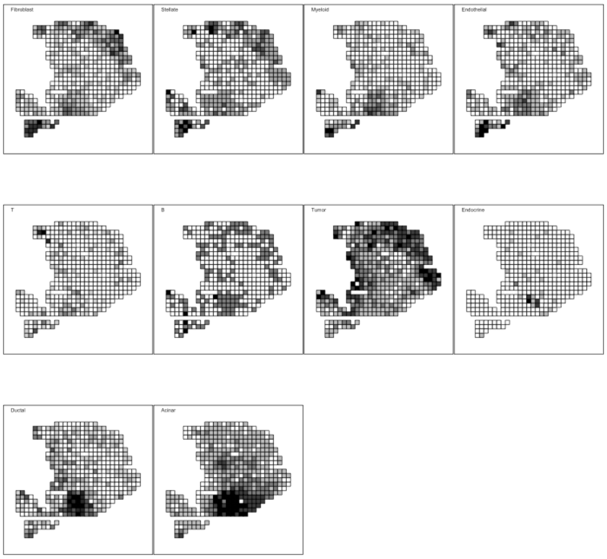

S2

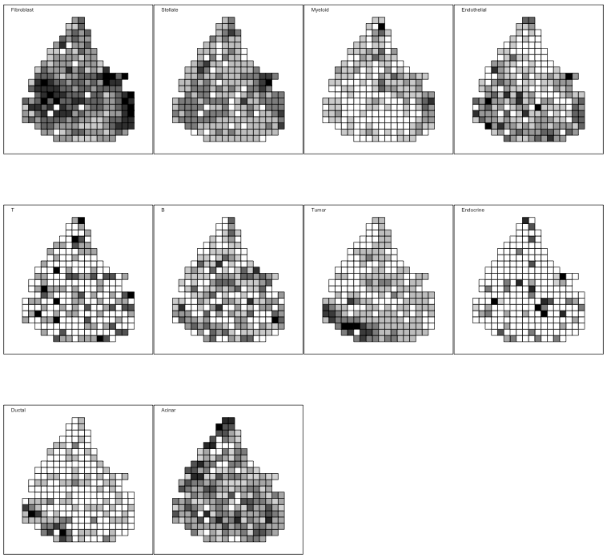

S4

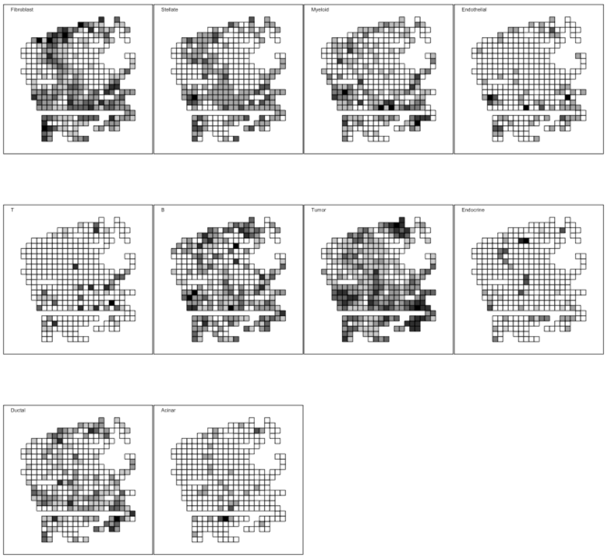

S5

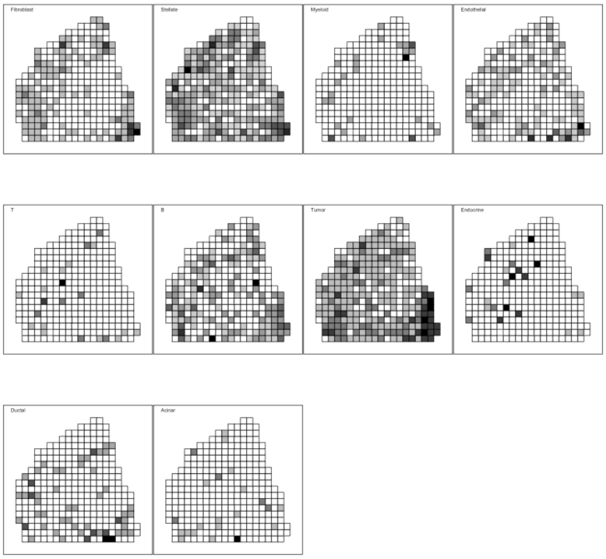

S6

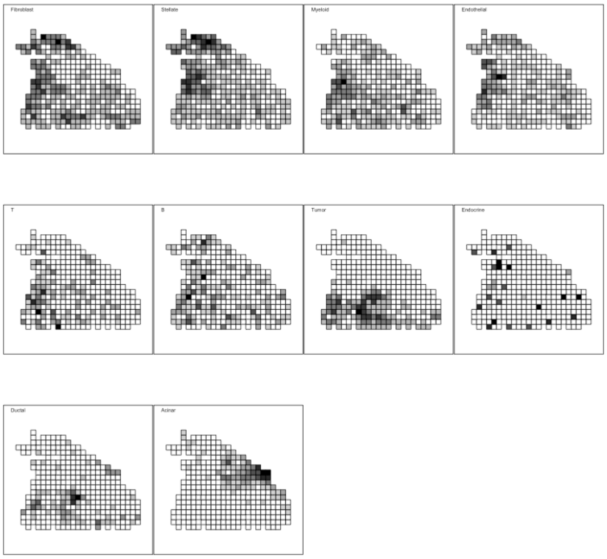

S7

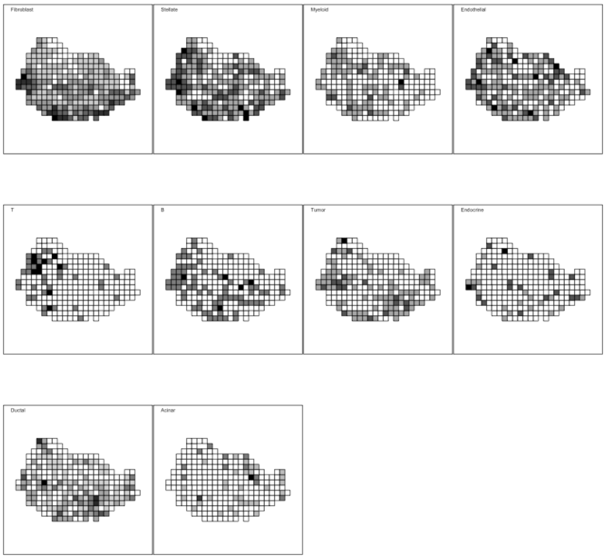

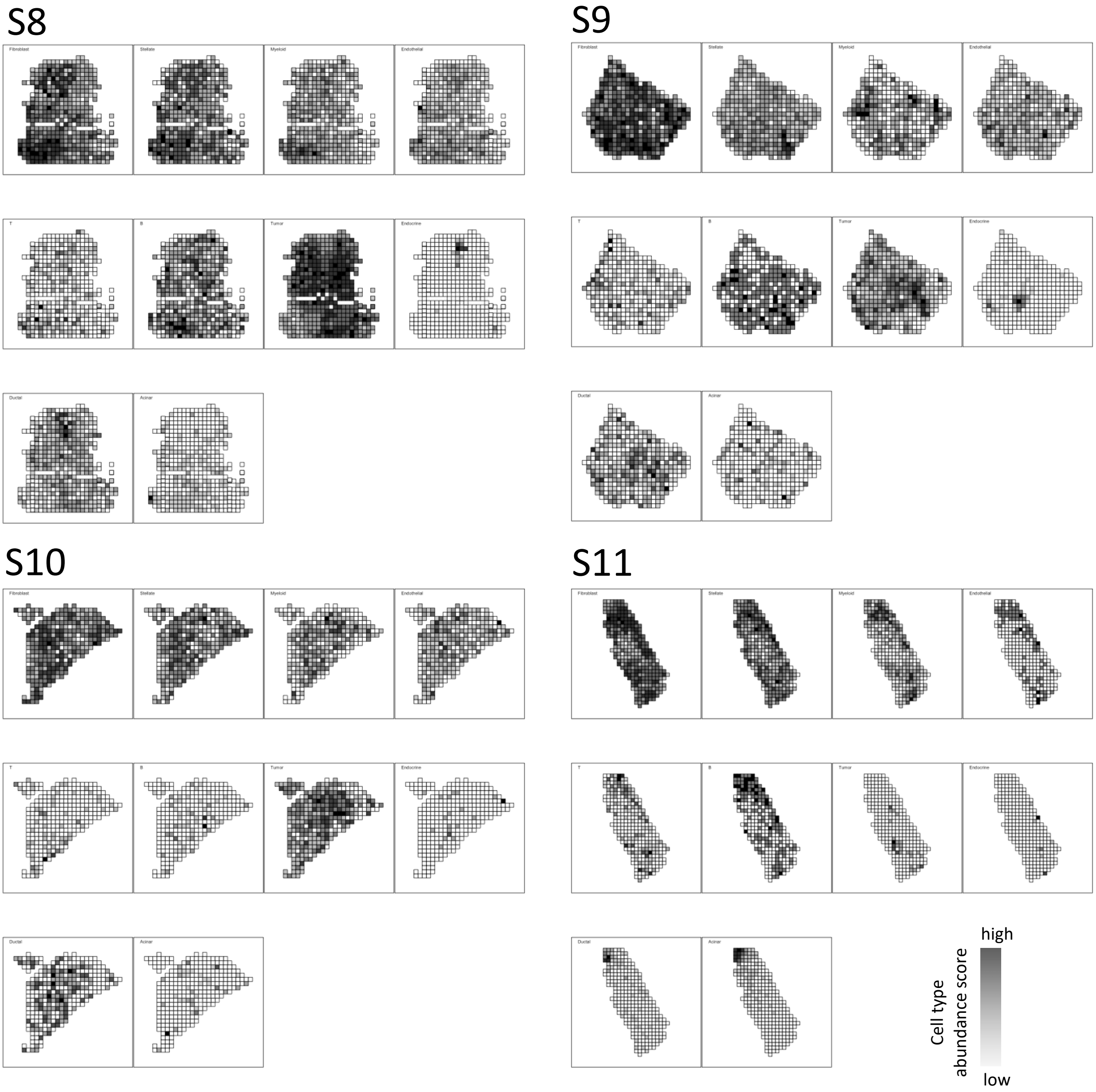

Supplementary Figure 1: Estimated cell type abundance scores for tumor, stromal, and immune cell types at the barcoded spatial locations in the histological slides of the pancreatic adenocarcinoma samples. Darker color indicates higher abundance in respective figures. See the Methods for details regarding the calculation of cell type abundance score.

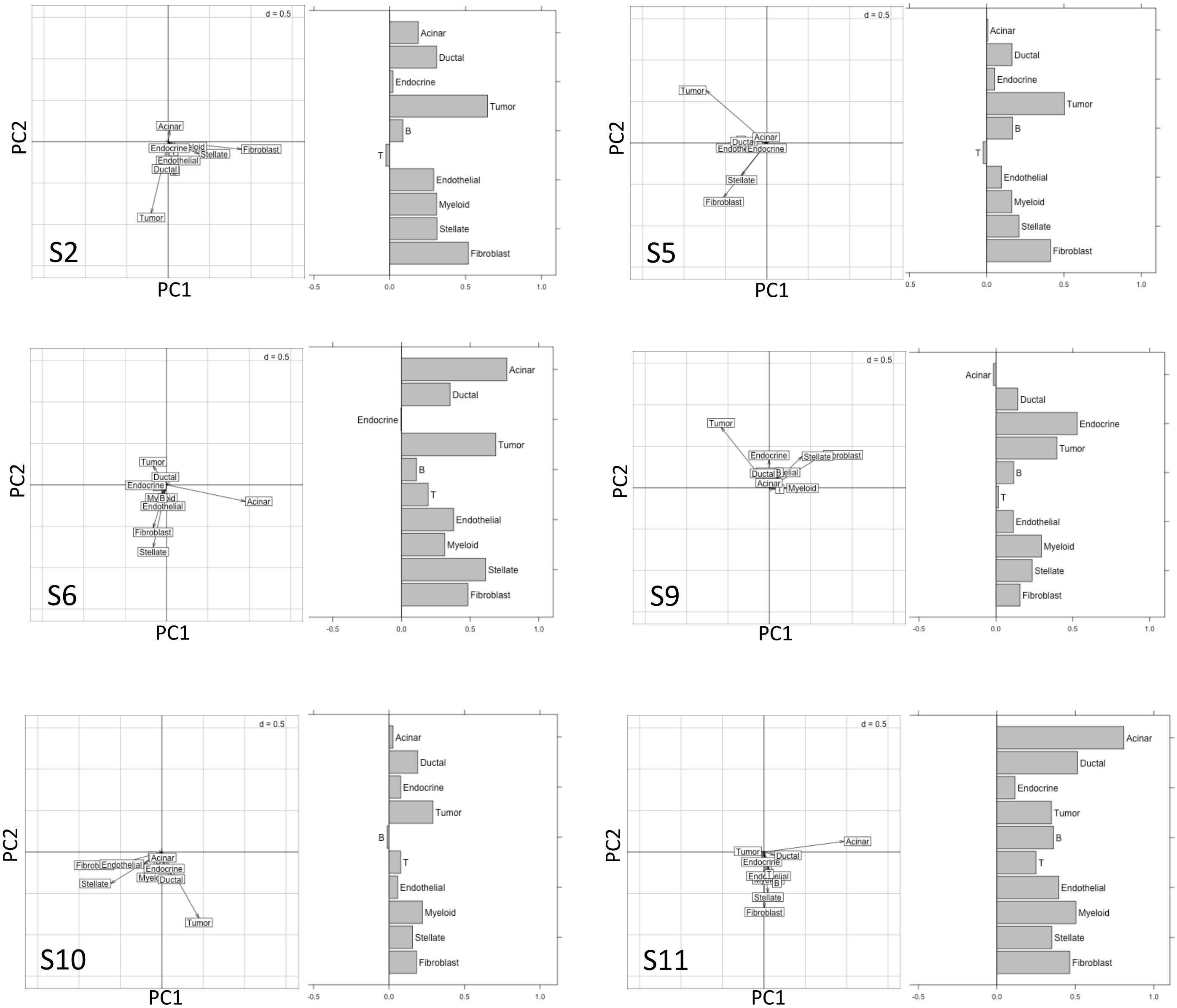

Supplementary Figure 2: Multivariate spatial analysis showing joint variation in spatial localization of the cell types in the pancreatic tumor samples (four samples are shown in main figures). Spatial PCA plot shows the loading of different cell types along the first two principal axes. Moran's I indicates the extent of spatial autocorrelation coefficients of the cell types.

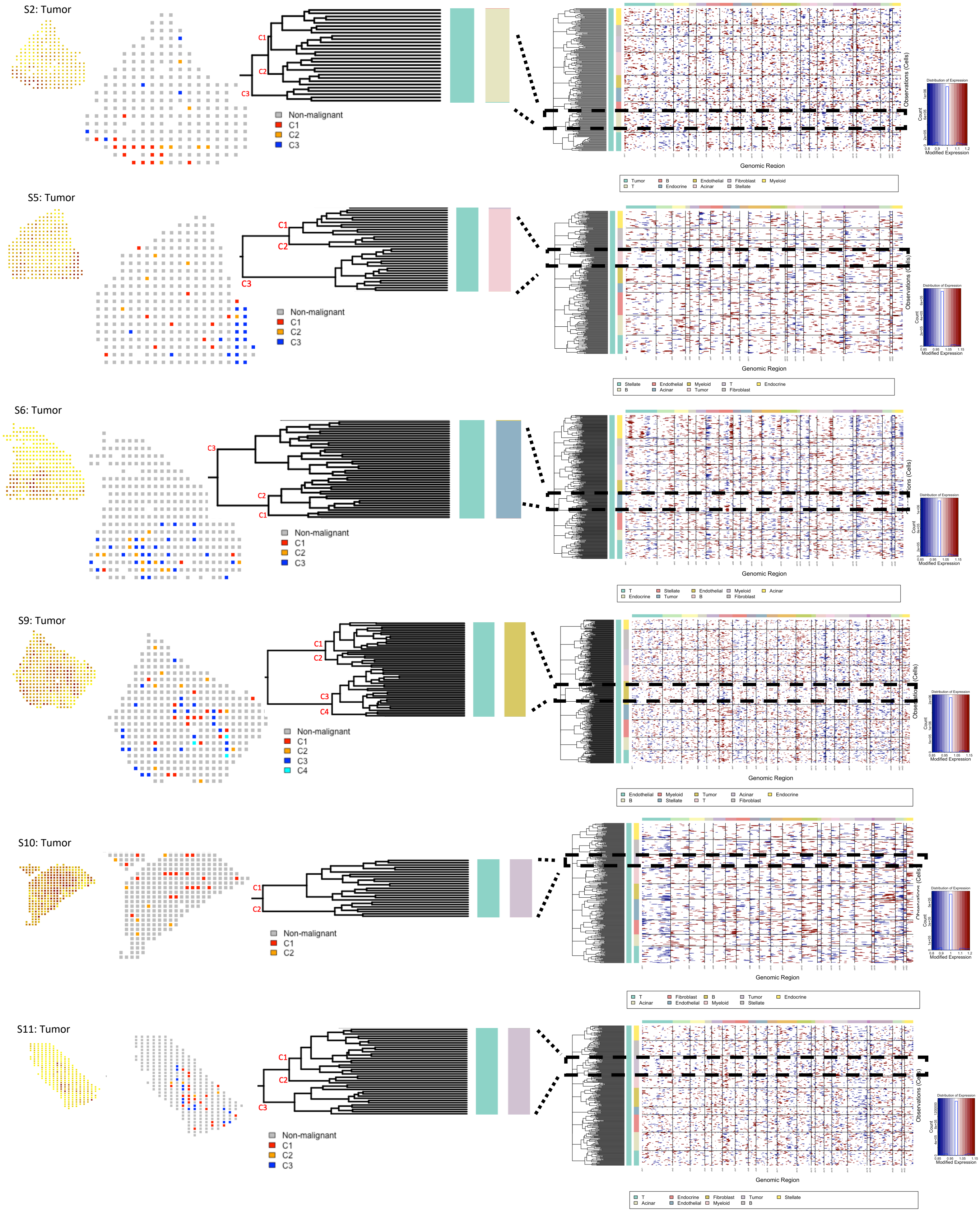

Supplementary Figure 3: Copy number profiles estimated from spatial gene expression data for pancreatic tumor samples (four samples are shown in main figures). Hierarchical clustering of cells in each of the pancreatic tumor samples based on copy number profiles estimated using InferCNV, with each row corresponding to a cell, ordered by cell types and clustered within each cell type by copy number patterns. Dashed-rectangle reflect tumor-specific patterns and the zoomed-in dendrogram shows main tumor subclones, with visualization of spatial localization of the subclones and corresponding tumor abundance areas.

S1

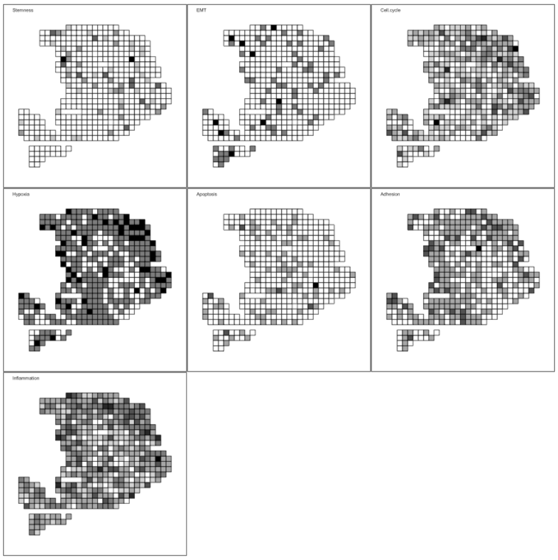

S2

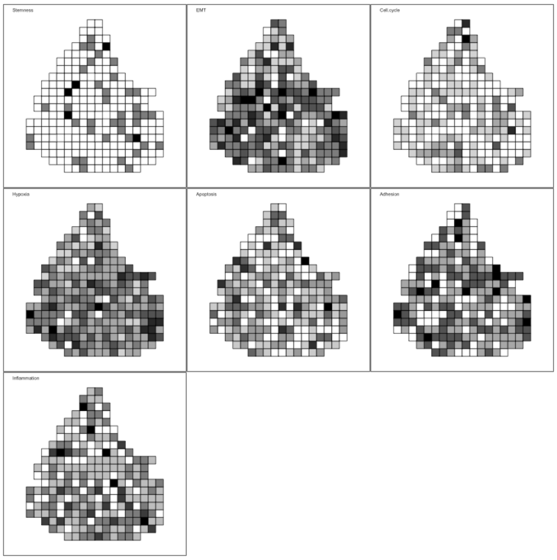

S4

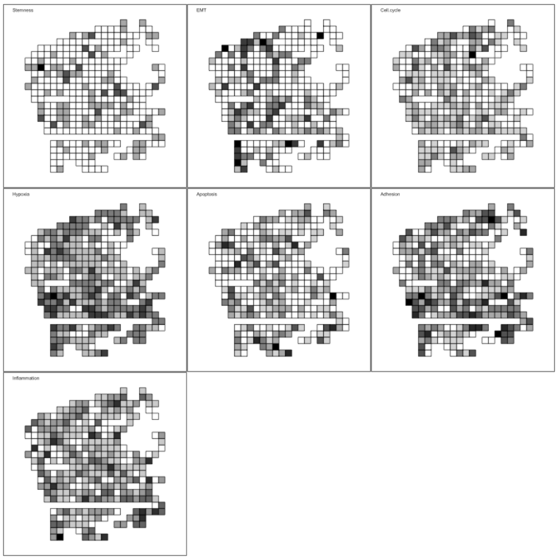

S5

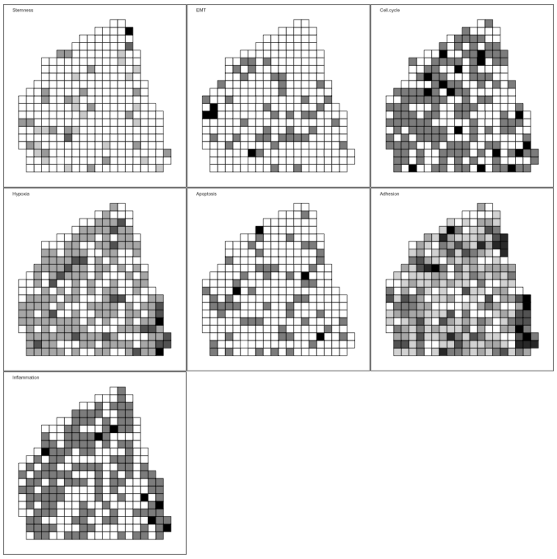

S6

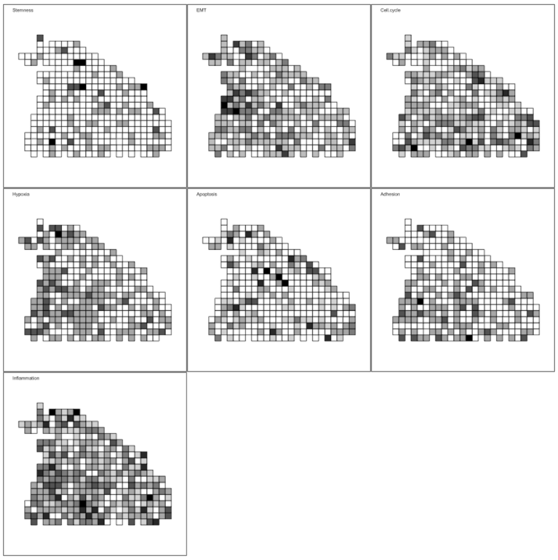

S7

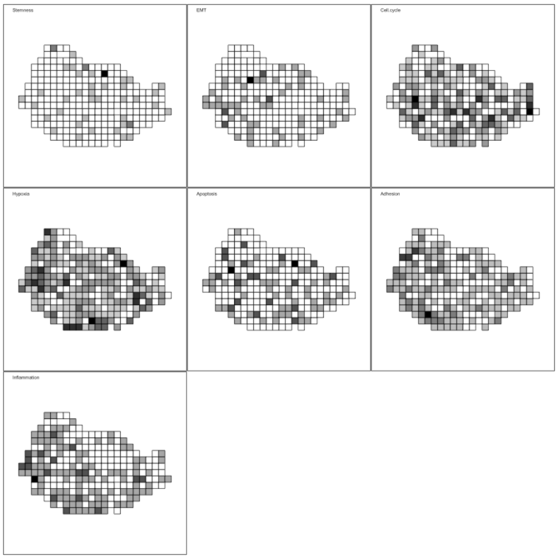

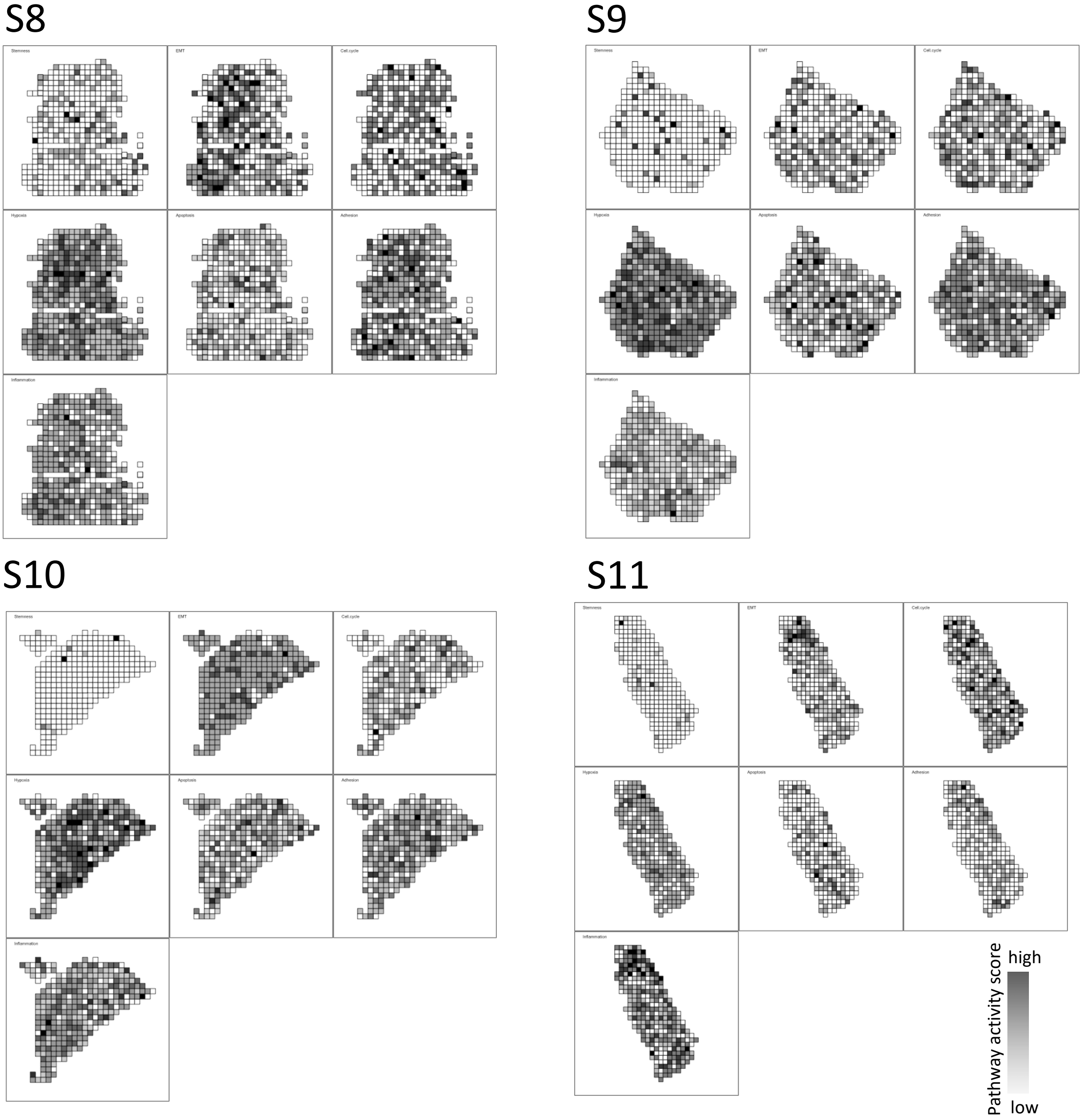

Supplementary Figure 4: Estimated activity scores for cancer hallmark related pathways at the barcoded spatial locations in the histological slides of the pancreatic adenocarcinoma samples. See the Methods for details regarding the calculation of pathway activity score.

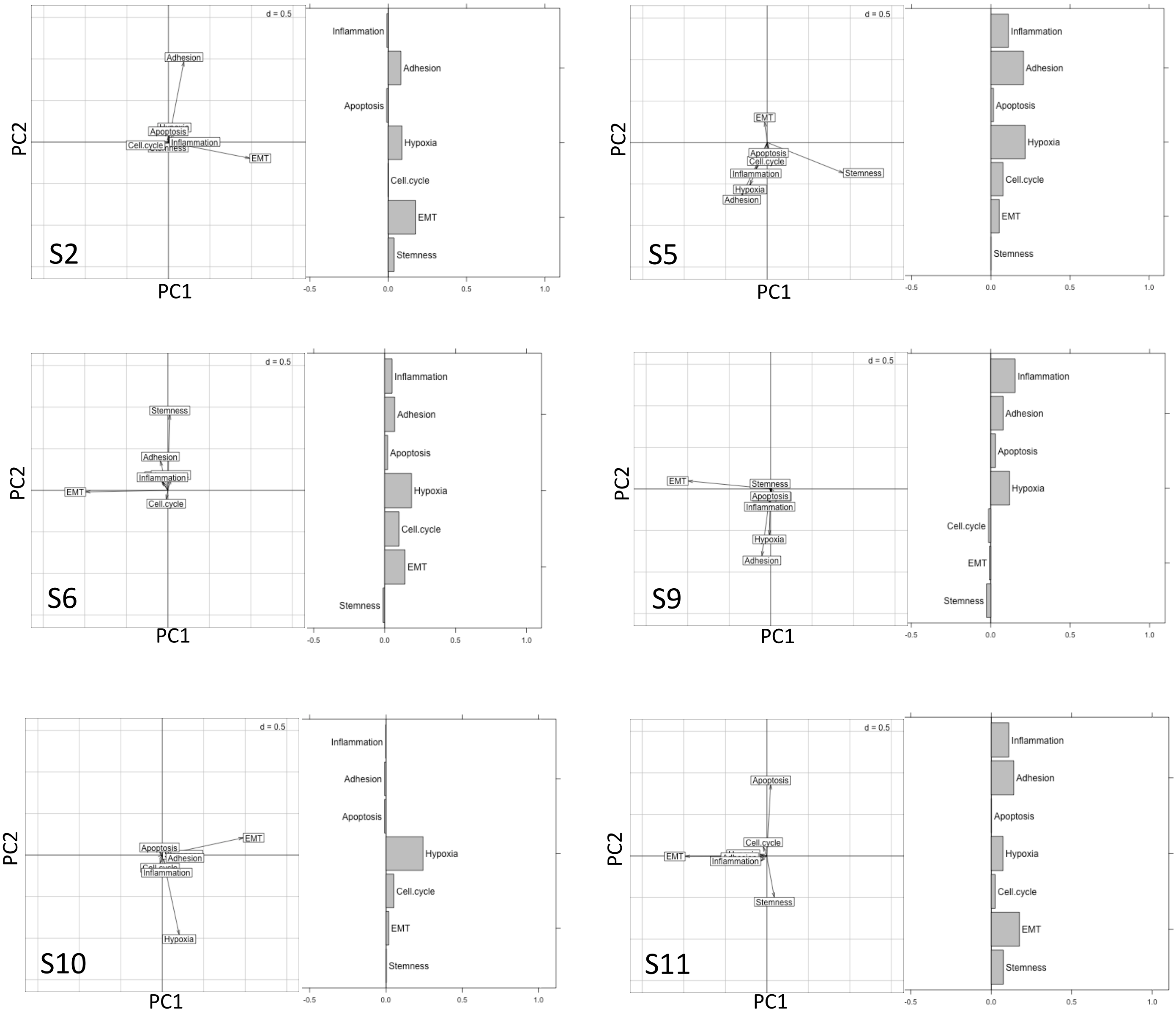

Supplementary Figure 5: Multivariate spatial analysis showing joint variation in spatial localization of the pathways associated with cancer hallmarks the pancreatic tumor samples (four samples are shown in main figures). Spatial PCA plot shows the loading of different pathway scores along the first two principal axes. Moran's I indicates the extent of spatial autocorrelation coefficients of the pathways.
